# What is the nature and impact of cognitive difficulties following hormonal treatments for prostate cancer?: An interpretative phenomenological analysis

**DOI:** 10.1007/s00520-024-08749-z

**Published:** 2024-07-22

**Authors:** Lorna Pembroke, Kerry A. Sherman, Haryana M. Dhillon, Heather Francis, Howard Gurney, David Gillatt

**Affiliations:** 1 Lifespan Health and Wellbeing Research Centre, Macquarie University, 2109 Sydney, NSW Australia; 2 School of Psychological Sciences, Faculty of Medicine, Health and Human Sciences, Macquarie University & Macquarie University Hospital, 2109 Sydney, NSW Australia; 3 Centre for Medical Psychology and Evidence-Based Decision-Making, School of Psychology, Faculty of Science, University of Sydney, 2006 Sydney, NSW Australia; 4 Psycho-Oncology Cooperative Research Group, School of Psychology, Faculty of Science, University of Sydney, 2006 Sydney, NSW Australia; 5 Macquarie University Clinical Trials Unit (CTU), Faculty of Medicine and Health Sciences, Macquarie University & Macquarie University Hospital, 2109 Sydney, NSW Australia; 6 Macquarie University Urology Clinic, Faculty of Medicine and Health Sciences, Macquarie University & Macquarie University Hospital, 2109 Macquarie Park, NSW Australia

**Keywords:** Prostate cancer, Cognition, Qualitative research, Cancer survivorship, Rehabilitation

## Abstract

**Objective:**

Prostate cancer hormonal treatments (e.g. androgen deprivation therapy) yield clinical benefits. However, there is increasing evidence these treatments may adversely impact cognitive functioning. This study aimed to qualitatively characterise the nature and impact of cognitive difficulties following these treatments.

**Methods:**

Prostate cancer survivors (PCS) self-reporting cognitive difficulties following hormonal treatments (via an online survey) and their partners were invited to participate in semi-structured interviews. Telephone or videoconferencing interviews were conducted, then transcribed, double-coded and analysed using the Framework Method, following the principles of Interpretative Phenomenological Analysis.

**Results:**

Eleven participants (six PCS and five partners) were interviewed. PCS reported a range of cognitive difficulties, verified by their partners, including forgetfulness, “fogginess”, fatigue and slowed processing speed. For some PCS, word-finding difficulties, tangential speech and memory problems were apparent during interviews. The aetiology of the reported cognitive difficulties was unclear as it was attributed to a possible combination of cancer treatments, compounding side-effects (e.g. fatigue, sleep problems, hot flashes), exacerbation of pre-existing conditions and/or age-related changes. Cognitive difficulties were reported to have led to shifts in self-perception, interpersonal dynamics and increased emotionality. Engagement in cognitively-stimulating activities and reliance on compensatory strategies were reported to be helpful in managing some cognitive difficulties. All participants endorsed the potential benefits of neuropsychological intervention.

**Conclusions:**

There are a diverse range of cognitive difficulties following hormonal treatments for prostate cancer experienced by PCS and their partners. Understanding the impact of these difficulties is important for the development of targeted neuropsychological interventions.

**Supplementary Information:**

The online version contains supplementary material available at 10.1007/s00520-024-08749-z.

## Background

Cancer-related cognitive impairment (CRCI) and its impact on individuals and families are gaining attention in cancer survivorship. In prostate cancer, one of the most common malignancies worldwide reported cognitive side-effects from hormonal treatments are not uncommon. Prevalence ranges between 10 and 69% of prostate cancer survivors (PCS) [1]. Hormonal treatments for prostate cancer involve suppressing production of androgens (e.g. testosterone, estradiol) to reduce tumour growth. This can be achieved by decreasing androgen production (e.g. luteinizing hormone-releasing hormone agonists, gonadotropin-releasing hormone agonists, orchiectomy, CYP17 inhibitors), and/or blocking androgen in the body (e.g. anti-androgens, androgen receptor antagonists) [2]. Hormonal treatments are often combined with other treatment modalities (radiation therapy, surgery, chemotherapy) and can be administered continuously or intermittently [2].

Increasingly research supports the role of sex hormones, such as androgens, in cognitive functioning [3]. Androgens and their metabolites have been demonstrated to have neuroprotective effects in maintaining cognitive functions [4]. Cognitive changes following hormonal treatments for prostate cancer often occur in the context of an ageing population already at risk of developing cognitive impairments (mean age at diagnosis is around 65 years), where a myriad of age- and treatment-related factors (e.g. biological, psychological, socio-environmental, cancer and accumulated toxicities from treatment) likely contribute to cognitive changes [5]. Other factors may include cancer stage, comorbid health conditions or impacts of hot flashes, fatigue, sleep disturbances and psychological morbidity (e.g. depression, anxiety, post-traumatic stress) [6].

CRCI is often mild to moderate in nature and can be transient or more persistent [7]. The complex interplay of aforementioned factors underlying CRCI in PCS likely contributes to reported brain fog and disruptions to attentional processes leading to declines in other cognitive domains such as memory and higher-order executive functions [8–14]. These cognitive difficulties may significantly impact quality of life, interpersonal, psychological, role and occupational functioning, as well as autonomy and safety, especially in older individuals [15]. Moreover, PCS on hormonal treatments often report increased emotional lability, irritability and anger [16]. Challenges with emotional and/or behavioural regulation likely impact relationship dynamics. However, little is known about any possible interaction between CRCI and relationship dynamics in PCS.

There is limited research exploring PCS experiences of CRCI following hormonal treatment and the impact of these changes on their lives and their families. This study aims to qualitatively explore the nature and impact of cognitive changes following hormonal treatments in PCS from the perspective of PCS and their partners.

## Methods

### Design

We used an inductive qualitative approach guided by principles of Interpretative Phenomenological Analysis (IPA) [17] applied with the “Framework Method” [18]. IPA is a participant-oriented approach recognising the double hermeneutical process. Since cognitive difficulties (e.g. word-finding problems, attentional or memory lapses) may be exhibited during interviews, the IPA approach facilitated the analysis of these behaviours. The Framework Method allowed for the integration of an inductive and deductive approach using a systematic method of organising and categorising data into a matrix. The Consolidated Criteria for Reporting Qualitative Research (COREQ) Checklist was used to guide the reporting of this study [19].

### Recruitment

Participants were recruited from a larger cross-sectional survey investigating cognitive functioning in PCS. This study was registered with Australian New Zealand Clinical Trials Registry on the 25th November 2021 (registration number ACTRN12621001608853). English-speaking PCS, aged 18 years and older, on hormonal treatments (e.g. androgen deprivation therapy) or on “watchful waiting”/ “active surveillance” were recruited from Australian clinics, healthcare professionals, email invitations, research registers (i.e., HealthMatch), social media (i.e., Facebook, Twitter), community groups and by word of mouth between January 21, 2021, and October 15, 2023. Participants receiving hormonal treatments, consented online to be contacted about participating in an interview and endorsed problems with cognition, were invited via phone call or email to participate in an interview. The criteria for identifying PCS with CRCI involved either endorsing “yes” to a list of treatment side-effects including “changes in thinking (e.g. memory, attention)”, or scoring ≥ 1 standard deviation below the mean of non-cancer controls on the Perceived Cognitive Impairments subscale from the Functional Assessment of Cancer Therapy—Cognitive Function (FACT-Cog) and/or the cognitive functioning scale from the European Organisation for Research and Treatment of Cancer Quality of Life Questionnaire (EORTC QLQ-C30). This conservative approach included PCS who might have milder cognitive difficulties compared to their similar age peers without prostate cancer. Partners were recruited through PCS and provided separate consent. Ethical approval was obtained from Macquarie University Human Research Ethics Committee (reference number: 52020611919011), in accordance with the NHMRC National Statement on Ethical Conduct in Human Research (Commonwealth of Australia, 2007- Updated May 2015) and the Declaration of Helsinki.

### Procedures

Semi-structured interviews were conducted by Author 1 in the participants’ preferred format. Participants selected interview via telephone or videoconferencing, which were audio-recorded. Interviews were semi-structured allowing flexibility in response-driven inquiry. Topics discussed included onset of cognitive changes, nature and impact of cognitive difficulties, coping and neuropsychological interventions (see Supplementary Materials). PCS reported on their own experiences, whilst partners of PCS provided their perspective on the PCS’s cognition.

Consent was confirmed verbally and recorded in the interview. PCS and their partners were interviewed separately, privately and only once, except for one couple who requested a joint interview. Notes and initial reflections were recorded both during and after each interview. As number of individuals suitable for interview was limited by recruitment from a cross-sectional survey and eligibility constraints, the concept of information power was used to determine when sufficient richness of data had been obtained and interviews stopped [20]. All interviews were transcribed by Author 1 and research interns. Transcripts and findings were not returned to participants for review.

### Analysis

Interview transcripts were coded with themes identified using both IPA guidelines and the Framework Method. Firstly, brief descriptive summaries of participants’ experiences of cognitive changes following prostate cancer treatment were produced for each transcript to allow the researchers to be “participant-observers”. Open coding was employed for each transcript, identifying concepts arising, making comments on language, repetition, contradictions, followed by phenomenological coding (i.e., line by line analysis). Tangential speech was coded as responses that were irrelevant to the questions asked or general topic of conversation, and/or identified by participants themselves as off-topic. After multiple readings of transcripts, concepts arising from initial steps were clustered into superordinate themes and subthemes. Research interns met with Author 1 to discuss the themes, identifying quotes to characterise and support themes. An experienced qualitative researcher (Author 2) oversaw the analysis. Data were compared across individual participants to develop a working then final analytical framework. Lastly, transcripts were recoded and data were charted into the framework matrix for interpretation. Any themes lacking rich evidence to add value to the emerging framework were eliminated. Qualitative data were managed and organised using N-Vivo 20 (QSR International, 2021). Methodological rigour was maintained through acknowledgement of reflexivity, team discussion and debriefing and iterative changes to the interview guide.

## Results

The sample consisted of 11 participants residing in Australia: six PCS and five partners (all female). An additional two partners did not wish to participate in the interviews due to time constraints or the topics being “too difficult to talk about”. Mean interview duration was 52 min, ranging from 20 to 71 min. The median age of our sample was 72 years (range 61 to 79), full demographic details and cancer characteristics of PCS are outlined in Table 1. Five themes were identified from the analysis, which are elaborated below (see coding tree in Supplementary Materials).

**Table 1:** Cancer characteristics of men with prostate cancer on hormonal treatments

ID	Age	Highest education level	Work status	Cancer treatments	Cancer stage	Time since diagnosis	Type of HT	Duration of HT	HT timing	Partner interviewed
PID01	73	Postgraduate degree or equivalent	Self-employed	RT and HT	Metastatic	3 months	Degarelix	12 months	Monthly injections	PID01-P
PID02	68	Completed Year 12	Retired	CT and HT	Metastatic	7 months	Leuprorelin	1 month	Four monthly	PID02-P
PID03	72	Undergraduate degree or equivalent	Retired	CT and HT	Metastatic	10 months	Goserelin acetate	11 months	Three monthly	PID03-P
PID04	75	Postgraduate degree or equivalent	Retired	CT, S, RT, HT	Metastatic	5 years 6 months	Goserelin acetate	24 months	Four monthly	PID04-P
PID05	61	Secondary education and vocational studies/training	Part-time	S, RT, HT	Locally advanced	1 year 9 months	Leuprorelin	24 months	Six monthly	-
PID06	69	Postgraduate degree or equivalent	Retired	CT, RT, HT	Metastatic	4 years 11 months	Bicalutamide and leuprorelin	4 years 7 months	?	-
PID07	79	Completed Year 10	Retired	S, HT, RT	Metastatic	6 years 8 months	Abiraterone acetate*	3 years 7 months	Four monthly	PID07-P

*This treatment was likely administered in combination with a gonadotropin releasing hormone agonist and not reported by the participants

*CT* chemotherapy, *HT* hormonal treatments, *RT* radiotherapy, *S* surgery.

### Theme 1: Diverse nature of cognitive difficulties and its challenges

Table 2 presents example quotations of the diverse nature of cognitive difficulties and their challenges. PCS reported a range of cognitive difficulties, verified by their partners, including difficulties with attention, memory, word-finding, organization, slowed processing speed and general cognitive decline. Most PCS described feeling “not as sharp” or more “foggy”, often associated with fatigue, slowed speed of processing, attentional problems and mistake-making. These interrelated difficulties were more pronounced during complex tasks requiring increased attentional processing and resulted in increased time and effort to complete familiar tasks.

**Table 2:** Quotes to illustrate the diverse nature of cognitive difficulties and its challenges

Subthemes	Quotation
General cognitive decline	“*My IQ had gone down and as such I couldn’t do the tasks that I did before […] It was more that my mind was not as sharp […] throughout my life I’ve always been able to do very quick responses. Sometimes I do a quick response and then I think to myself, oh my god, what did I say?*” (PID02)
More pronounced difficulties on attention-demanding task	“*My ability to concentrate for any period of time on on a complex subject has deteriorated. […] even now if I, if I watch a movie on the TV in it, and the storylines get all complex, I really got to think about it*.” (PID06) “*I just finished a fairly complex analysis task for the Owners Corporation, but it took me a week to do that and even then, you know the result was okay, and they’re very happy with it but I don’t think it was as good as what I used to be able to do*.” (PID06)
Proneness to making mistakes	“*I was reading in an email that he sent to somebody, he had used the wrong something—he got the names wrong and it’s very unlike him. I mean, I don’t think– prior to this, he would never have any mistakes on his emails because he normally reads them and re-reads them, so yeah… He was paying less attention to details in a way.*” (PID02-P)
Inefficient or unreliable encoding of information	“*I don’t grab on to things the way I used to be. A lot of stuff just sort of flows by.*” (PID06) “*He might remember it, but he might get it a bit mixed more than anything.*” (PID07-P)
Relying on prompting from partners	“*I have to remind him of things […] I am feeling that I have to do a lot more.”* (PID01) *“He’s just got to be reminded all the time.*” (PID03-P)
Repetition required to compensate for deficits	“*I had to re-read and then re-read and then read again and then think about it and basically to get a firm grasp on what it was.*” (PID02)
Word-finding difficulties and its impact	“*I’ll be talking, and about something, and I’ll go to mention a word, and it’s gone.*” (PID06) “*I don’t have the same grip on language*” (PID04) “*I’m delayed on my normal fluid speech. It’s delayed by pauses while I look for the word*.” (PID01) “S*ometimes I sort of picked the word out and then I think to myself, “Well, that’s not really what I meant*.” (PID03)
Increased disorganisation	“*Now he’s very disorganised like every time he’s gone doing something, I’ll come and he’ll say, “I’ve lost a bulb.” The amount of times we’ve lost parts of cars that we’ve had to go and buy and eventually it’s like…. minor things he’ll get it going, but at the moment now got a lawn mower pulled apart. I’ve got a tractor pulled apart, a caravan, you know? That’s what’s happening*.” (PID07-P)
Increased tangentiality	Around 19% of PID01’s transcript was coded as tangential speech, with seven incidences of stating “*I just had another senior moment. What was the question again?*”
Driving concerns and decreased confidence	*“He is losing confidence, he’s definitely, because I’m doing all the driving.”* (PID01-P) *“My mother worries all the time now and look PID07 is driving. I watch all the time though […] I always go I. I would never trust him to go, even for an hour to [town]”* (PID07-P)

Difficulties with short-term memory and “forgetfulness” were prominent concerns for participants. This was characterised by inefficient or unreliable encoding of information. Some men employed external strategies to aid their memory or often relied on their partners to prompt and remind them. Moreover, repetition was necessary to compensate for deficits in information processing and poor retention.

Word-finding problems were reported by most PCS and were observed during interviews. This often disrupted the flow of conversation and impacted expressive meaning.

Few PCS reported higher-order executive function problems (e.g. problem-solving, decision-making, planning), except for increased disorganisation. Qualitatively, some PCS demonstrated tangential speech during the interview, on several occasions forgetting the question asked.

Confidence and concerns about driving were also raised by both PCS and their partners, with partners assuming increased responsibility in driving and/or helping the PCS navigate.

### Theme 2: Uncertainty about aetiology of cognitive difficulties

Quotations supporting the following responses regarding the aetiology of cognitive difficulties are presented in Table 3. Since many PCS received a combination of treatments, most expressed uncertainty as to whether their cognitive changes were solely due to hormonal therapy or compounded treatments and their side-effects (e.g. fatigue, hot flashes, sleep problems). Hormonal treatments were also perceived as exacerbating pre-existing conditions. PCS acknowledged difficulty distinguishing between “normal” age-related processes and treatment-related cognitive decline, as well as the potential of treatment exacerbating the normal ageing process. Furthermore, treatments may have increased awareness of cognitive changes.

**Table 3:** PCS beliefs about the aetiology of cognitive difficulties

Subthemes	Quotations
Uncertain casual relationships	“*Whether it was the hormonal injection or whether it’s the chemotherapy, I have no idea, but most certainly there were side-effects in terms of my cognitive… my thinking in terms of being muddled.*” (PID02)
Compounding treatment side-effects	“*I don’t feel as sharp as I used to be, if you like, so now, do you put that down as tiredness or if I wasn’t tired, would it still be not as sharp as it as it would have been if you like? […] this feeling of not being as sharp or as quick, is that the drugs or tiredness, don’t know?*” (PID05)
Interaction of side-effects and comorbidities	Both PID04 and PID04-P reported pre-existing sleep problems from restless leg syndrome, sleep apnoea, and anxiety, along with current treatment-side effects (e.g. fatigue and hot flashes) that may contribute to cognitive difficulties and fatigue: “*When I haven’t slept, I I I I don’t think very well and I I don’t function very well.*” (PID04)
Possible contribution of normal ageing	*“My memory as well is not as good as it used to be and I don’t know if that’s age or one of the sorts of side effects of this treatment.”* (PID06) *“I have no idea whether it’s associated with my age or with my treatment.”* (PID04)
Exacerbation of age-related decline	“*ADT is exacerbating and intensifying a normal negative age-related occurrence and the deterioration in motion and physical mobility and energy levels and, and all those chemical/ biochemical things that make one of a particular gender* […] *it’s exacerbating normal aging deterioration and with respect to cognition, fatigue.*” (PID01)
Greater awareness of difficulties	“*He is a little bit more forgetful but noticing it more himself.*” (PID01-P)

### Theme 3: Shifts in self-perception, interpersonal dynamics and increased emotionality

Table 4 presents example quotations supporting the following expositions of this theme. Difficulties with cognitive functioning were reported to lead to shifts in self-perception. A loss of confidence, and poorer quality of life and daily functioning were reported by PCS. Decreased confidence, fatigue along with cognitive side-effects were reported to lead to increased social withdrawal and potentially leaning more into pre-existing traits (e.g. introversion). For instance, PCS indicated perceptions of accelerated cognitive decline led to decreased engagement in professional settings and at family gatherings.

**Table 4:** Quotes to illustrate shifts in self-perception, increased emotionality and interpersonal dynamics

Subthemes	Quotations
Shifts in self-perception	*“I just don’t feel like myself.*” (PID05)
A loss of confidence, and poorer quality of life and daily functioning	“*I’m getting confused, and I can’t remember facts, and it’s a hell of a way to live and I don’t want to live this way. Yes. One of the reasons I’m stopping ADT at four months— I will never have any more— is because the quality of life to me, the joy of life, is damaged […] I resigned my position on the latest paper as corresponding author, because I didn’t feel confident to engage in technical discussions with the editor […]. The editor wants to talk to me, but I’ve declined to do that because I’m afraid I’ll make some silly mistakes and jeopardise the paper publication.*” (PID01)
Social withdrawal	*“I won’t go to that group of friends, PID04-P will go by herself and I’ll I’ll stay home because I don’t feel well enough and confident enough to go to it.”* (PID04) “*I’m becoming a little reluctant to engage in conversation. I was at a family gathering at last weekend and I barely said boo. I just sat at the table and listened. No, in the past I would be taking an active part but I’m becoming a sort of a more of a hermit and becoming less and less engaged with life […] I tend to draw in my borders and limit my daily activity and limit my social involvement with the world […] I have less and less interest and friends and family*.” (PID01)
Leaning more into pre-existing traits	“*He’s been more introverted than me always, so that’s a factor in this I think, and he’s more living by that introversion, rather pushing himself to be sociable.*” (PID04-P)
Partners experiencing increased responsibilities	“*You just sometimes feel that you are taking on a lot more responsibility and have to think for more than yourself.*” (PID03-P)
Shifts in power dynamics	“*Before he used to have the upper hand in everything, but now it’s just gone totally the other way round.*” (PID03-P)
Greater appreciation for partners	“*I found him nicer. As he’s- as [husband] has got sicker, I suppose you’d say, and he relies on me so much more, he’s been a lot, a lot nicer person.*” (PID07-P)
Increased irritability and emotional lability, which impacted interpersonal dynamics	“*He tends to have less boundaries around what he says to people. […] I think a major thing would most likely be the difficulty in managing emotions, the emotional lability that comes with the hormone treatment […] if you’re you're more easily reactive, then you’re going to get more easily agitated, more easily angry, more easily tearful, and so on […] Depression as it does with everybody spills over onto other people. He would be more readily critical of me. More easily irritable.”* (PID04-P) “*He is definitely more emotional than he used to be, as in a young bloke was here and we hadn’t seen him for four years and they and he went back and [PID07] cried, which [PID07] would have never cried before, but he did.*” (PID07-P) “*Personally think the drug has made me probably just that that more reactive sometimes to some comments or what’s going on than I would have been before.*” (PID05)

The cancer experience and cognitive difficulties were reported to lead to shifts in relationship dynamics, especially interpersonal interactions and responsibilities between PCS and family members, which were reported by all partners interviewed. The sentiment of “*I am doing more things, I’m doing a lot more things*” (PID01-P) was present in many partner accounts, “doing more” included more driving, providing reminders, and household tasks. Such changes sometimes shifted “power” dynamics and led to greater appreciation for their partners. Moreover, several participants reported increased irritability and emotional lability, which impacted interpersonal dynamics.

### Theme 4: Coping and management

Table 5 presents quotations supporting the following range of different coping and management strategies employed by PCS. No PCS sought professional help for their cognitive difficulties, instead cognitive difficulties were managed by engaging in cognitively stimulating activities and compensatory strategies. Most PCS were proactive in "*keeping busy*” and keeping their brain “*active*” with a range of cognitively stimulating activities, including establishing a men’s community organisation called “Men’s Shed”, being involved in building management, house and garden maintenance, managing paperwork for a band, writing sermons, engaging in casual work, socialising, and engaging in a “brain training” programme. Using external aids, such as lists and a diary, were the primary compensatory strategy used to aid memory, planning and organisation. However, consistent implementation of external aids was necessary to support cognition. Interestingly, some reported changes in attitude, becoming more relaxed, as a means of coping with cognitive changes.

**Table 5:** Range of coping and management strategies employed by PCS

Strategies	Quotations
Coping with cognitive changes	
Brain training	“*I started getting back into an only very recently and they’re very simplistic things but it’s like speed, adding and subtracting and putting in things. There’s a Brain Age thing in there… some of those programs, so I’ve started redoing that just because it’s quick and easy and rather simplistic, but I you know I enjoy doing those things and the main reason I started, I think, this must be helping my brain a little bit.”* (PID05)
External compensatory aids	“*I notice that he’s doing a lot more “to do” lists, and he previously didn’t because it would have just been up there.*” (PID02-P) “*I have to write things down even more closely as I have no great grasp of what’s going to happen the next few days.*” (PID01)
Effectiveness of external aids depending on consistency	“*The only thing is I don’t think he always remembers to write it down, and also I mean I’m still working so I’m not here all the time.*” (PID03-P)
Relaxed attitude to cognitive changes	“*These days, I seem to be a bit laid back about it, you know don’t bother with the details, just let’s get to the results sort of thing*.” (PID06)
Coping with cancer more broadly	
Antidepressant medication	“*I also take an antidepressant and I’m not sure what—I take this, what’s it called? Venlafaxine and I’m not sure if it is that, or it’s just me, but I think I’m much calmer than I used to be.*” (PID06)
Engaging in health promoting behaviours	“*I completely changed my diet taking myself into a much more healthier diet and the big thing is is [sic] that I gave up drinking.*” (PID02)
Allied health support	*“[Prostate cancer nurse] has been my angel, and she guided me and supported me and given me better advice than I’ve got from anyone else at [hospital].*” (PID01)
Cancer centres in providing holistic care	“*The Prostate Cancer Centre in [city] is an amazing facility and they are extremely helpful if I need …*” (PID04)
Information-seeking	“*I’m a little bit of a Fact Finder. So, you know, Mr. or Mrs Google, and take that with a grain of salt, but you know what sites, and they’re all just, you know, the cancer- The Cancer Council stuff. So [wife] printed out all their brochures on prostate cancer.*” (PID05)
Social support	“*We have friends and they ring quite often, and our family ring quite often to keep in touch*.”(PID03-P)
Benefit of pets	*“It has been the best thing, and I’ve found that his mood has improved a lot, and his personal… he is feeling himself, he’s a lot happier, sweet talking the dog and, you know, just like better in himself.*”(PID01-P)
Keeping positive both mentally and behaviourally	“*I was very cognizant of keeping my mindset positive […] the last thing that I wanted around me was people being all mopey and depressed and sad and all that kind of thing. I really try to stay away from them […] I do not want negative people around me because that would affect my positiveness.*” (PID02) “*It’s not trying to trick the brain, it’s just while I’m up there- if you act happy, be happy, then you are happy, if it sort of makes sense. If you’re there and whinging and carrying on and the sky is falling, then you’re not going to feel that crash hot, in my opinion, because everything’s all doom and gloom.*” (PID05)

PCS also employed a range of strategies to cope with the impact of cancer more generally, including antidepressant medication and engaging in health promoting behaviours. Support from allied health professionals was highly valued in providing holistic cancer care such as prostate cancer nurses. Cancer centres were helpful in providing holistic care. Information-seeking was another coping behaviour employed by both men and their partners in managing the impact of cancer. Participants also reported the benefits of social support from family, friends and community groups and pets. Keeping positive was another coping mechanism reported involving both mental and behavioural processes.

### Theme 5: Opinions on a neuropsychological group intervention

Table 6 presents quotations supporting the following opinions of a neuropsychological intervention from PCS and their partners. Whilst PCS received support for physical side-effects of treatment, there was little support for the cognitive side-effects. All participants affirmed the potential benefits of a group neuropsychological intervention, if cognitive rehabilitation/remediation were possible.

**Table 6:** Opinions on a neuropsychological intervention

Subthemes	Quotations
Little support for the cognitive side-effects	“*Any literature that I’ve read and or doctors have said to me, they had they focus more on the physical side, not the mental side from keeping it sharp […] If you are forgetting stuff, is there a program or something to help you with that? […]. Going to the gym will help your bones, doing exactly x y z here will help with your brain.”* (PID05)
Potential benefits and interested in a group neuropsychological intervention	“*If it seems that it is possible that it could improve my memory situation, then that would be very helpful*.” (PID03) *“If I honestly believe that there were solutions to the issues that I face, I would be interested.*” (PID01)
Need for a roadmap to encourage a positive direction	“*It needs to be a very positive positive idea, it can’t be allowed to devolve into a bunch of victims. […] If you’re feeling sorry or depressed about it—done. There’s no point, it’s not going to change anything. Yeah, it’s just kind of going to make your life worse, your family’s life worse. You know you been given a second chance, grab it and go with it.*” (PID06)
Contribution from men going through similar experiences	“*Might be more receptive coming from somebody who’s going through the same thing as them rather than a medical expert or specialist.*” (PID02)
Personalised, patient-centred and PC-relevant	“*This is YOUR diagnosis. This is YOUR treatment. This is YOUR medications, here’s the information about YOUR medication. […] Instead of just getting a brochure that’s written for everybody Joe Blow that you think—doesn’t apply to me, doesn’t apply to me, don’t read the rest. If they think that applies to them, that would be very helpful to the family as well.”* (PID01-P) “*Strategies and I would see on my part that would prompt you to do things or reminders of some kind.*” (PID03-P)
Qualities of a good facilitator	“*Someone who’s a good listener because you need to listen to people and, and someone who can who’s got a good empathy with people. If you can’t relate to them, then it’s not going to work.*” (PID06) “*They need to learn good group work skills. They need to be able to sit with people who are in distress and link them up, connect them up with one another to work with the sociometry, the connections between group members because that’s what will support them and help them feel understood […] running it [the group] in a mindful way […] A cohesive, positive group culture where people are free to express anything that’s going on in them. Not just the intellect. […] making it both safe and inviting and welcoming […] if you don’t bring it back to the present moment you missed a lot.*” (PID04-P)
Benefits of a smaller group or buddy system	“*Four to six would be excellent*.” (PID04-P) *“I think for a lot of men especially old men have grown up, born a baby boomer generation, mine, are not terribly comfortable talking about disease and illness. […] I would say a buddy system for men my age is better than group discussions*.” (PID01)

Participants shared a range of ideas potentially important for development of a neuropsychological intervention. These included the need for a “*roadmap*” to encourage a positive direction and contribution from PCS going through similar experiences. Information should be personalised and patient-centred. In addition, strategies to aid cognitive functioning should tailored to the concerns noted by PCS and family members.

Qualities of a good intervention facilitator included being a good listener, empathetic and having group work skills to create a positive, safe, welcoming space. However, there was some apprehension towards having a “large” group, with a smaller group or “buddy” system being more desirable.

## Discussion

Our results highlight the nuanced and diverse range of perceived cognitive difficulties experienced by PCS undergoing hormonal treatments. They indicated impacts on self-perception, daily functioning and interpersonal dynamics. There was general interest and specific feedback regarding the potential for a neuropsychological intervention, suggesting PCS and their partners would engage with such an intervention.

The diverse nature of cognitive difficulties and their associated challenges reflect previous research. Decline in attention, processing speed, verbal/language skills, memory, visuospatial and visuomotor skills and executive functioning have been identified in PCS on objective neuropsychological tests following hormonal treatments [8–13]. Although findings have been inconsistent [21, 22], this suggests a subset of PCS may be more vulnerable to experiencing cognitive changes following hormonal treatments. Moreover, as our qualitative data demonstrate, cognitive decline may remain undetectable to others despite self-reported challenges of PCS in participating in daily activities (e.g. driving, social engagement) and reduced productivity [23]. Reported difficulties on more cognitively demanding tasks add to earlier findings indicating hormonal treatments likely disrupt complex information processing (i.e., using higher-order executive processes) rather than specific cognitive domains [14]. Such deficits may manifest more evidently in ecologically valid contexts (e.g. work environment) over standard neuropsychological testing conditions. This is important given cognitive complaints may increase the likelihood of early retirement in older cancer survivors [24]. Therefore, addressing cognitive complaints may play an important role in effective return to work and optimising productivity, impacting both personal wellbeing (i.e., quality of life, mental health and financial stability) and societal economic health more generally [25].

Our respondents were unclear regarding the aetiology of cognitive changes following hormonal treatment, as these changes were co-occurring with other potential causal factors including other cancer treatments, ageing, co-morbid illnesses and psychological factors (e.g. depression, anxiety, stress). For instance, ageing-related decreases in testosterone levels have been associated with decline in memory functioning, processing speed, visuospatial skills and visuomotor abilities in older men [26], which are consistent with neuroanatomical areas containing androgen receptor expression (hippocampus, amygdala, etc.) [27]. Development of CRCI is likely multifactorial [28] associated with the cancer itself [29], cancer treatments, exposure to hormonal treatments [30], genetic susceptibilities and other medical comorbidities [31]. Cancer and its treatments have also been associated with chronic neuroinflammation, DNA-damage, endothelial dysfunction, all increasing risk of brain ageing [32]. Moreover, our data highlighted the perceptions of accelerated brain ageing following treatment, which support preliminary neuroimaging data [33]. Additionally, we explored how clusters of co-occurring, interrelated symptoms (e.g., hot flashes, sleep problems, insomnia, fatigue and depression) may lead to cognitive deficits. Causal relationships, however, are currently speculative, requiring further investigation. Furthermore, older individuals are likely more sensitive to declines in androgen levels, treatment-related toxicities and disturbances in physical and psychological functioning, increasing the risk of CRCI [5].

PCS in our study reported on the impact of treatment side-effects on self-perception and emotionality that in turn affected relationship dynamics. Novel insights into the perceived impact of cognitive or intellectual functioning on self-image in PCS were identified in our sample of highly educated men, who may have been more sensitive to decrements in cognitive performance. Our data suggest such decrements occurred along with decreased self-confidence, which accompanied deficits in occupational functioning and social withdrawal, leading to shifts in interpersonal relationships. Whilst couples commonly report role shifts after a cancer diagnosis, changes in role functioning (e.g. employment, independence, personal responsibilities) may challenge core beliefs (e.g. self-image and perceptions of masculinity) [34]. The nature of relational shifts and extent to which they are a consequence of CRCI requires further investigation, especially as partners of PCS are likely part of an ageing population experiencing normative cognitive decline and potentially having challenges of their own. Moreover, whilst treatment side-effects like urinary incontinence have been found to lead to social isolation in PCS [35], our results provide a unique perspective of the impact of cognitive side-effects, consistent with research more broadly on cognitive decline and social withdrawal [36]. Our data also highlighted how changes in interpersonal relationships may be further complicated by increased emotional lability following treatment. Thus, neglecting to address the impact of hormonal treatments on partners and the dyadic relationship may diminish the physical and psychological wellbeing of PCS [37].

Despite all participants acknowledging the potential benefits of a neuropsychological intervention, none had sought professional help for their cognitive difficulties. This was partly due to a lack of information provided by treating professionals and limited availability of interventions, consistent with reviews of the research [15]. Lower rates of help-seeking for PC-related concerns have also been reported in the literature with around 40% of PCS not seeking support from health professionals or support services [38]. Moreover, 82% of men reported unmet supportive care needs with 47% reporting unmet psychological needs [38]. Factors associated with lower help-seeking included older age, low cancer knowledge, elevated depressive symptoms, embarrassment, fear and adherence to masculine norms (e.g. self-reliance, stoicism, appearance of control) [39, 40]. Addressing barriers to help-seeking is pivotal, given the apparent conflict between perceived supportive care needs and help-seeking behaviours in PCS. Some solutions presented by Cowan et al. (2023) include encouraging healthcare providers to facilitate conversations around CRCI with PCS, identifying people at risk of developing CRCI, conducting assessment of CRCI and providing therapeutic support and management of these symptoms [15]. 

Regarding the management of CRCI, we found PCS applied range of compensatory strategies (e.g. note-taking), but their level of success varied. Enhancing the effectiveness of these strategies may involve neuropsychological interventions like cognitive rehabilitation. Whilst there is growing evidence supporting the benefits of cognitive rehabilitation in cancer populations [41], its effectiveness in PCS has not been determined. It is possible broader emotional or psychological support will also assist in coping with CRCI. Additionally, prostate cancer nurses can play a crucial role in connecting individuals to these supports and may also help overcome reluctance to help-seeking [42].

Our study has several limitations. Whilst the use of qualitative methodology allows for in-depth exploration and understanding of participants’ experience, it is characterised by self-selection bias. The sample primarily consisted of retired, older, Caucasian men with advanced prostate cancer, potentially increasing their susceptibility to CRCI due to treatments effects, associated with its type and duration, and tumour metastases [28]. Moreover, younger PCS still in the workforce may have different experiences and needs compared to current participants, highlighting the need for further research in this area. There were also no participants treated only with hormonal therapies, which meant we could not tease out the cognitive impacts of hormone therapies alone. Though perceived cognitive functioning often poorly correlates with objective measures and is more closely associated with psychological factors [43, 44], it may better reflect the day-to-day experiences of cancer survivors compared to standardized neuropsychological measures and tend to be a stronger predictor of quality of life and functional outcomes [45].

PCS may experience a diverse range of cognitive difficulties. Their aetiology is likely multifactorial. Cognitive difficulties may negatively impact self-perception, daily functioning and interpersonal dynamics. Whilst neuropsychological interventions addressing the cognitive, psychological and behavioural concerns may be of benefit, it is critical to incorporate current approaches to working therapeutically with older males to increase engagement of PCS with any intervention.

### Supplementary Information

Below is the link to the electronic supplementary material.

Supplementary file1 (DOCX 84 KB)

## Data Availability

No datasets were generated or analysed during the current study.
