# Supplementary material for: What is the nature and impact of cognitive difficulties following hormonal treatments for prostate cancer?: An interpretative phenomenological analysis
Source: Support Care Cancer. 2024 Jul 22;32(8):534. doi: 10.1007/s00520-024-08749-z (PMC11263254; doi:10.1007/s00520-024-08749-z)
Supplement: Supplementary file 1 — Supplementary file1 (DOCX 84 KB) [file 520_2024_8749_MOESM1_ESM.docx]

**Supplementary Material**

This document contains:

1. Semi-structured interview guide
2. Reflective statements from the authors
3. Coding tree

Semi-structured Interview Guide

| **Cognitive problems** |
| --- |
| What are your main concerns with your thinking?   - Ask for examples - Impact on life (e.g., have these difficulties prevented you from doing some of the things you would like to do?) - Impact on family life, social life, work? Driving? Future plans? Finances? ADLs? - Can you pinpoint when it’s generally more worse? - When did you or others first notice these concerns? (before/after treatment)     You mentioned how you have difficulties ______ after commencing treatment, did you also notice changes to:   - Memory (e.g., taking in info, forgetting, needing cues to remember) - Working memory (e.g., keeping track of finances, mental calculations) - Attention (easily distracted? Multi-tasking) - Executive functioning (organising, planning, problem-solving, figuring ways to judge different demands of life) - Processing speed (taking you slower to do things) - Language (word-finding, any difficulties understanding or expressing yourself) - Visuospatial (e.g., navigating – reading maps, orienting yourself around the place) - Personality? (e.g., more irritable etc.?) |
| **Mood/sleep** |
| - Cognitive changes impacting mood - Mood impacting cognition - History of low mood/anxiety? Ever received a diagnosis or seen a psychologist? - Sleep |
| **Coping** |
| What are some things you do to help manage your cognition? Mood?   - Social life? - Activities to keep brain active? - Activities to improve mood?     Sharing concerns with treating doctor or prostate cancer nurse? |
| **Future support** |
| - Have you seen anyone about your concerns with your cognition? - What kind of support would you like? - Thoughts on neuropsychological intervention? |
| **Is there anything else you think would be important for us to know?** |
|  |

**Reflective Statement for Author 1**

I am a clinical neuropsychology registrar, registered with Australian Health Practitioner Regulation Agency (AHPRA), and a PhD candidate under the supervision of the co-authors of this paper.

I have prior experience conducting qualitative research in psycho-oncology working with breast cancer populations. In my clinical role, I have experience in conducting interviews on both patients and their partners addressing the nature and impact of cognitive difficulties in a diverse range of conditions (e.g., mental health, neurodegenerative, neurological and developmental) for both diagnostics and rehabilitative purposes. I have come across prostate cancer survivors struggling with cognitive difficulties following cancer treatment in my clinical private practice work (none of these clients have been recruited for this study).

Being a younger woman, I may not fully appreciate the challenges older men with prostate cancer may face. However, I will do my best to open-minded and curious about participants’ perspectives and experiences. I do want to acknowledge that several of my family members have been diagnosed with cancer, two of them affected by prostate cancer. This has driven my interest in survivorship concerns and supportive care interventions.

No relationships with any of the participants were established prior to the study’s commencement. Participants were aware that this study was part of my PhD study.

**Reflective Statement for Author 2**

I am a Professor of Health Psychology and a health psychologist registered with the Australian Health Practitioner Regulation Agency (AHPRA), and the primary supervisor of the PhD candidate leading this research.

I have extensive prior experience conducting qualitative research across a range of health-related contexts, with a particular focus on psycho-oncology, including lymphoedema. This includes conducting interviews with patients addressing a range of aspects related to treatment decision making, coping and quality of life. As a middle-aged woman I acknowledge that I may not be fully aware of the difficulties and challenges faced by men diagnosed with prostate cancer. However, having experienced people who I know well being diagnosed with this disease has given me a range of insights as to the potential for cognitive difficulties to arise that may be associated with prostate cancer treatment. I approach this qualitative research with an open mind, and cognizant of my own experiences and beliefs when partaking in the analysis process.

No relationships with any of the participants were established prior to the study’s commencement.
